# *In Vivo* and *In Vitro* Metabolites from the Main Diester and Monoester Diterpenoid Alkaloids in a Traditional Chinese Herb, the *Aconitum* Species

**DOI:** 10.1155/2015/252434

**Published:** 2015-02-03

**Authors:** Min Zhang, Chong-sheng Peng, Xiao-bo Li

**Affiliations:** School of Pharmacy, Shanghai Jiao Tong University, 800 Dongchuan Road, Shanghai 200240, China

## Abstract

Diester diterpenoid alkaloids (DDAs), such as aconitine (AC), mesaconitine (MA), and hypaconitine (HA), are both pharmacologically active compounds and toxic ingredients in a traditional Chinese herb, the *Aconitum* species. Many DDA metabolism studies have been performed to explore mechanisms for reducing toxicity in these compounds and in *Aconitum* species extracts for safe clinical administration. In this review, we summarize recent progress on the metabolism of toxic AC, MA, and HA and corresponding monoester diterpenoid alkaloids (MDAs) in the gastrointestinal tract and liver in different animal species and humans *in vivo* and/or *in vitro*, where these alkaloids are primarily metabolized by cytochrome P450 enzymes, carboxylesterases, and intestinal bacteria, which produces phase I metabolites, ester hydrolysed products, and lipoalkaloids. Furthermore, we classify metabolites detected in the blood and urine, where the aforementioned metabolites are absorbed and excreted. Less toxic MDAs and nontoxic alcohol amines are the primary DDA metabolites detected in the blood. Most other DDAs metabolites produced in the intestine and liver detected in the urine have not been reported in the blood. We propose an explanation for this nonconformity. Finally, taking AC, for instance, we generalize a process of toxicity reduction in the body after oral AC administration for the first time.

## 1. Introduction

Diester diterpenoid alkaloids (DDAs, Table 1), such as aconitine (AC), mesaconitine (MA), and hypaconitine (HA), are a family of highly toxic alkaloids from the root of a traditional Chinese herb, the* Aconitum* species (sp.), which has been used clinically for years. Monoester diterpenoid alkaloids (MDAs, Table 1) are the ester hydrolysis products of DDAs at the C-8 position, which are also components of this herb. Both DDAs and MDAs exhibit excellent pharmacological effects, including anti-inflammatory, analgesic, and cardiotonic activities [1, 2].

However, these compounds, especially DDAs, have narrow therapeutic windows. For example, a single lethal AC dose for humans is estimated at 2–6 mg [3, 4] with poisoning symptoms, such as hypotension, palpitations, ventricular tachyarrhythmias, asystole, and numbness of the face and limbs [1]. Severe poisoning may occur after improper ingestion of DDA-containing drugs or prescriptions, such as Chuanwu [5], Caowu [6], and Fuzi [7]. Therefore,* Aconitum* herbs are traditionally boiled or steamed before oral administration to ensure safety [8]. During this process, DDAs are mainly hydrolysed to less toxic MDAs. Further MDA hydrolysis yields almost nontoxic alcohol amines (Table 1), such as aconine, mesaconine, and hypaconine [3, 9, 10]. In contrast with AC, the half-maximal lethal dose (LD_50_, mg/kg, i.v. mice) of 14-benzoylaconine (BAC) and aconine increases by approximately 38- and 430-fold, respectively [11].

On the other hand, many valuable studies have recently been performed on DDA and MDA metabolism to explore the toxicity reduction mechanisms and obtain information for clinical guidance. In this paper, we review for the first time the metabolites biotransformed in the gastrointestinal tract and liver from toxic AC, MA, and HA of DDAs as well as their corresponding ester hydrolysed products, BAC, 14-benzoylmesaconine (BMA), and 14-benzoylhypaconine (BHA) of MDAs, in different animal species and humans* in vivo* and* in vitro*. Furthermore, we classify the metabolites detected in the blood and urine, in which these metabolites are absorbed and excreted. Our study will be fundamental and helpful for further studies on reducing the toxicity of DDA-containing drugs compatible with other medicine based on DDAs absorption and metabolism [12, 13].

## 2. Metabolism in the Gastrointestinal Tract and Liver

Traditional Chinese prescriptions are commonly prepared through decoction and ingested orally. The active compounds are unavoidably converted in the gastrointestinal tract.

### 2.1. Metabolism in the Stomach

The stomach provides an acidic environment for drug dissolution and absorption; however, studies on stomach metabolism are typically ignored. Only one study has focused on AC metabolism in the stomach.

In this study, 14 metabolites and 2 ester hydrolysis products are identified in gastric content in rabbits after oral AC administration [14]. Metabolism includes hydroxylation, deoxylation, demethylation, didemethylation/deethylation, and ester exchange at the C-8 position with long chain fatty acids (Table 2). The enzymes responsible for metabolism have not been reported. The aforementioned metabolic process may be catalysed by CYP2C9 and CYP2C8 that are expressed in parietal gastric cells [15] and by bacteria that are located in the human stomach [16].

The ester hydrolysis products at the C-8 and C-14 positions are not only observed in rabbit stomachs but also in acid solutions (negative control). Ester hydrolysis in the stomach may be catalysed by carboxylesterases (CEs) in the gastric mucosa [17] because CE expression has also been reported in the stomach, although CEs are predominantly distributed in the liver, plasma, and intestine [18]. However, this finding also implies that DDAs can be nonenzymatically ester hydrolysed under acidic conditions, which is discussed in Section 5.

In addition, AC, MA, HA, and their hydrolysis products (MDAs and alcohol amines) are detected in gastric contents in a dead female, who was suspected of dying from acute drug poisoning involving* Aconitum* alkaloids [19]. However, the reference did not indicate whether the hydrolysis products were metabolized from DDAs in the stomach or were originally in the toxicant.

### 2.2. Metabolism in the Intestine

A large number of bacteria populate the gastrointestinal tract; the bacterial concentration increases distally. The majority of bacteria reside in the colon, where the density approaches 10^11^-10^12^ cells/mL, and anaerobic species dominate. This microbiota secretes a diverse array of enzymes that participate in various metabolic processes, such as reduction, hydrolysis, deoxylation, acetylation, deacetylation, and N-demethylation; thus, the intestinal microbiota is important to orally ingested drug metabolism [20, 21]. Notably, hydrolysis catalysed by bacteria is common in glycosides. Based on DDA and MDA structures, ester hydrolysis is likely driven by CEs, which also dominate the intestine [18].

The intestinal bacteria DDA metabolism reviewed herein was mainly performed* in vitro* through anaerobic incubation in a feces suspension, which included high levels of intestinal bacteria. The intestinal bacteria DDA metabolism is similar to metabolism in the stomach and included hydroxylation, deoxylation, demethylation, demethylation with deoxylation, ester hydrolysis at the C-8 and/or C-14 position, and ester exchange at the C-8 position with short and long chain fatty acids (Table 3). AC metabolites, such as 16-O-demethyl AC, 3-deoxy AC, and 16-O-demethyl-3-deoxy AC, were further converted to deoxylation, demethylation, ester hydrolysis, and ester exchange products (Table 4). These results imply that MDAs, which are DDA ester hydrolysed products, may be metabolized through the same pathway; however, no studies have reported on intestinal MDA metabolism.

Ester exchange metabolites are classified as lipoalkaloids or lipoaconitines with an acetyl group at the C-8 position of DDAs replaced by other fatty acid acyl groups [24, 31]. Presumably, the short chain fatty acids (such as propionic, butyric, hexanoic, phenylacetic, and phenylpropionic acids) for ester exchange are generated from xenobiotics, such as food decomposed by intestinal bacteria, while certain long chain fatty acids (such as palmitic, oleic, and stearic acids) are generated from bacterial cell walls [24]. DDA toxicity is reduced after ester exchange. For example, the LD_50_ of 8-O-butyryl- (from short chain fatty acid) benzoylmesaconine is 15.78 mg/kg, which is 5.5-fold greater than MA (8-O-acetyl-benzoylmesaconine) [22]. The LD_50_ for mice with lipomesaconitines (from long chain fatty acids) are from 10 to 40 mg/kg, which are 20-fold greater than MA [32].

### 2.3. Metabolism in the Liver

The liver is an important organ for drug metabolism, and it expresses many drug-metabolising enzymes. After oral administration, drugs are typically subjected to hepatic metabolism, including CEs that catalyse ester hydrolysis [18], phase I drug metabolic enzymes that catalyse oxidation, and phase II metabolic enzymes that catalyse conjugation [21]. The metabolites are hydrophilic and are more rapidly excreted from the body than parent drugs. Cytochrome P450 enzymes (CYP450s) and uridine 5′-diphosphate (UDP)-glucuronosyltransferases (UGTs) are the most common phase I and phase II metabolic enzymes, respectively [33].

The hepatic metabolism studies reviewed herein were mainly performed* in vitro* through incubation with liver microsomes. CYP450- or UGT-catalysed metabolism in microsomes can be selectively performed in different reaction systems with auxiliary enzymes and exclusive substrates [34, 35].

The DDA and MDA phase I metabolic pathways are similar and include hydroxylation, deoxylation, demethylation, didemethylation/deethylation, dehydrogenation, and demethylation with dehydrogenation (Table 5). The individual CYP450s responsible for specific metabolites were further determined via individual inhibitors or recombinant isoenzymes. CYP3A4 and CYP3A5 are the most common isoenzymes that catalyse both DDAs and MDAs. In addition, CYP2D6, CYP1A1/2, CYP2C9, CYP2C8, CYP2C19, and CYP2E1 also partially catalyse DDAs.

Hydrophobic drug biotransformation commonly occurs first through phase I metabolism in which functional groups, such as hydroxy, sulfhydryl, carboxyl, and amino group, are formed and provide reaction sites for the subsequent phase II conjugation [46, 47]. For lipophilic DDAs and MDAs, hydroxy groups are initially present and are formed after hydroxylation during the phase I metabolism. However, phase II metabolites of either DDAs or MDAs were not detected in hepatic metabolism* in vitro* and* in vivo*, which demonstrates that phase II metabolism is not dominant compared with phase I metabolism in the liver. DDA ester hydrolysis should be catalysed by CEs. However, CYP3A, CYP1A1, and CYP1A2 are also involved in ester hydrolysis of AC, which reflects the complexity of metabolism.

### 2.4. A Comparison of DDA and MDA Metabolism in the Gastrointestinal Tract and Liver

The metabolites generated in the stomach, intestine, and liver are compared in Table 6. The polarity of most metabolites increased after DDA gastrointestinal and hepatic metabolism, except lipoalkaloids. Metabolites of AC from dehydrogenation and demethylation with dehydrogenation were only observed in the liver. The AC metabolites from demethylation with deoxylation observed from intestinal bacteria incubation [24] were also detected in the urine after oral AC administration in rabbits. However, these metabolites were not found in the urine after intravenous injection [48]. This observation suggests that the gastrointestinal tract may participate in biotransformation. The characteristic metabolites in the gastrointestinal tract were lipoalkaloids, which might be converted by enzymes that are only produced by intestinal bacteria. In addition, more lipoalkaloid varieties were detected in the intestine than in the stomach, which is consistent with abundant bacterial distribution in the gastrointestinal tract [16]. More studies have focused on DDAs than MDAs. However, it is speculated that MDAs may share similar metabolic pathways (except for ester hydrolysis at the C-8 position) with DDAs in the gastrointestinal tract based on the similarity in their hepatic metabolism and chemical structures.

Interestingly, phase I metabolites of hydroxylation, deoxylation, demethylation, and didemethylation/deethylation were detected not only in the liver but also in the gastrointestinal tract. As mentioned above in Section 2.2, intestinal bacteria participate in metabolism, such as through deoxylation, reduction, and deacetylation. However, it has also been reported that human small intestinal epithelial cells express a range of P450s, which include CYP3A, the isoenzyme that dominates in the liver [49]. Intestinal metabolism was performed* in vitro* through anaerobic incubation in a feces suspension, despite the symbiotic intestinal bacteria, which should also contain apoptosis-undergoing intestinal epithelial cells that release phase I and phase II metabolic enzymes into the suspension. Thus, intestinal metabolites are likely converted by both bacteria and phase I metabolic enzymes.

Metabolic isoenzyme expression is not identical among different species [50] that lead to metabolic differences in different species. Based on references in this review, we find that DDAs were ester hydrolysed to MDAs in rat intestine and liver, but not in humans. On the other hand, the same metabolites converted in different species have been reported. For example, 16-O-demethyl BAC, the ester hydrolysed products from 16-O-demethyl AC in intestinal metabolism, was detected not only in rats but also in humans. Hydroxy aconitine from AC was detected through incubation in liver microsomes or S_9_ from humans, rats, guinea pigs, and mice. It is notable that the AC demethylation at the C-16 position is catalysed by CYP3A and CYP1A1/2 in rats while it is catalysed by CYP3A, CYP2D6, and CYP2C9 in humans. However, no studies have specifically compared metabolites from DDAs or MDAs among humans and different experimental animals. Briefly, the metabolic differences in different species yield certain risks in predicting human drug metabolism based on data from experimental animals.

**Figure 1:**
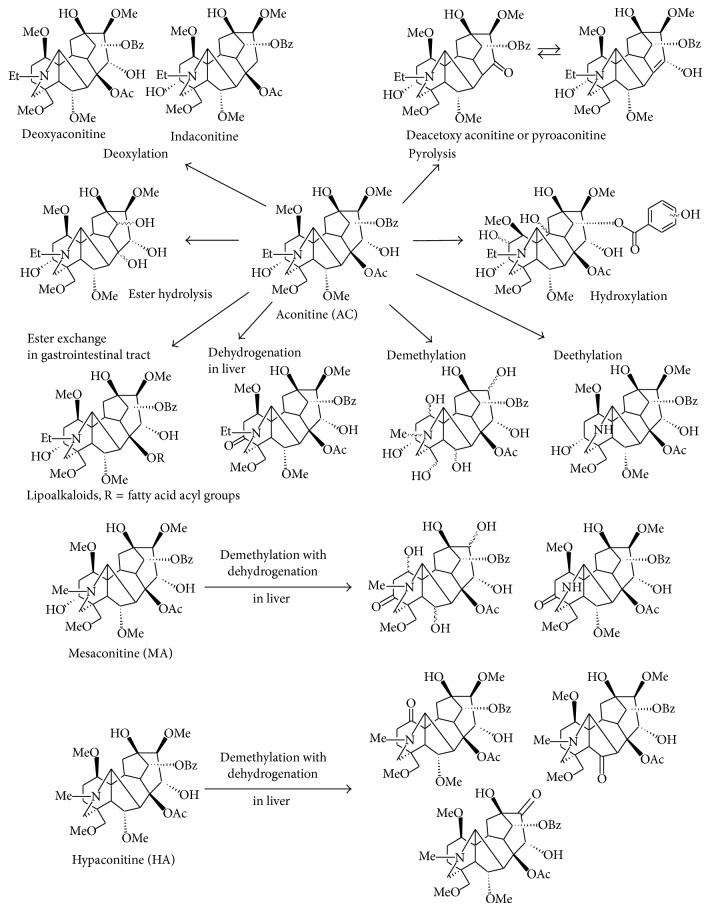
Proposed DDA metabolic pathways. The organ/tissue metabolic processes are partially indicated. The wavy bonds indicate the potential metabolic positions. Me, Et, Ac, and Bz indicate methyl, ethyl, acetyl, and benzoyl groups, respectively.

The metabolic pathways proposed for DDAs are generalized in Figure 1.

The organ/tissue metabolic processes are partially indicated. The wavy bonds indicate the potential metabolic positions. Me, Et, Ac, and Bz indicate methyl, ethyl, acetyl, and benzoyl groups, respectively.

## 3. Metabolites Detected in the Blood

MDAs and alcohol amines are the main DDA metabolites in the blood (Table 7). It has been suggested that AC and related alkaloids can be rapidly absorbed by the upper gastrointestinal tract for the short latent period between the ingestion of aconite roots and the onset of poisoning features [3]. Therefore, the absorbed DDAs may be partially and gradually ester hydrolysed to less toxic MDAs and nontoxic alcohol amines by CEs distributed in the blood. Furthermore, the blood provides a suitable pH environment for ester hydrolysis. This hypothesis is supported by an analysis of rat plasma after DDA administration via a tail vein, wherein MDAs and alcohol amines were detected [39].

MDAs and alcohol amines are commonly considered markers in forensic and clinical evaluations of aconitine poisoning because their half-lives are longer than DDAs [19], which might lead to the neglect of other metabolites in the blood. Additionally, many efflux/influx transporters, such as P-glycoprotein (P-gp), multidrug resistance-associated protein 2 (MRP2), and MRP3 expressed in intestinal epithelial and hepatic cells, are involved in drug absorption [53]. It is difficult to determine whether the various metabolites produced in the gastrointestinal tract and liver are transported into the blood from the few studies on their transport mechanism.

## 4. Metabolites Detected in the Urine

The metabolites found in the urine are shown in Table 8. Compared with intestinal and hepatic metabolites, most metabolites from hydroxylation, deoxylation, demethylation, deethylation/didemethylation, dehydrogenation, ester hydrolysis, deacetoxylation (pyrolysis), and demethylation with deoxylation have been found in the urine. Further, a few phase II metabolites as glucuronide and sulfate conjugates have been found in the urine but have not been reported in hepatic or intestinal metabolism* in vitro*. Glucuronidation catalysed by UGTs occurs in human and rat kidneys [63, 64]; glucuronidation might be responsible for phase II biotransformation processes in addition to hepatic and intestinal metabolism.

Additionally, mRNA for CYP3A4 and CYP3A5, which are the major isoforms that catalyse DDA metabolism, is also expressed in human kidneys, but the expression levels are much lower than in the liver and intestine [65]. Based on the data in Section 3, metabolites from DDAs in the blood are fewer than in the urine. Further, the urine is converted from the blood in the kidney. Perhaps, the various metabolites in the urine are converted from DDAs and their ester hydrolysed products in the blood by metabolic enzymes expressed at low levels in the kidney. Is it possible that various metabolites from DDAs produced in the intestine and liver are absorbed in the blood and excreted in the urine? However, as noted in Section 3, the data on metabolites in the blood is insufficient.

No studies have reported on metabolites of lipoalkaloids in the urine, which are the metabolites characteristically produced in the gastrointestinal tract. DDA lipophilicity may be reasonably increased through ester exchange with long chain fatty acids at the C-8 position, which results in easier absorption of lipoalkaloids into the blood. Are the ester groups then hydrolysed by CEs in the blood and liver, producing MDAs and alcohol amines, or are they directly excreted through the feces? Such conjecture requires further investigation.

## 5. Original Compound Stability

All of the* in vivo* and* in vitro* metabolism reactions occur in fluid. Therefore, the stability of DDAs and MDAs in different pH aqueous solutions should be considered. One study reported that AC and MA were decomposed dramatically after incubation in water for 24 h at 25°C (degrees Celsius), and the products of AC were BAC, aconine, deacetoxy AC, and deoxy AC. In addition, almost half of the AC and MA were depleted in phosphate buffer at pH 2.0 and 6.8 over 12 h at 25°C (degrees Celsius); these pH values are similar to gastric acid and intestinal juice, respectively [66]. These results imply that metabolites, such as BAC and aconine, may be partially converted from DDAs in body fluid without enzyme catalysis. On the other hand, the rate of MDA formation from DDAs was much higher in phosphate buffer (pH 7.4) with hepatic microsomes than in the negative control without hepatic microsomes [39]. The facts imply that the enzymes did affect bioconversion of instable DDAs.

## 6. Metabolite Detection and Identification

Metabolites are typically varied at trace levels with endogenous interference from biological matrices, such as tissue, the blood, or urine. Liquid chromatography multiple-stage tandem mass spectrum (LC/MS^*n*^) has been widely applied for drug metabolite detection due to its high sensitivity and selectively.

For DDAs and MDAs, positive electrospray ionization (ESI^+^) is suitable for alkaloid ionization. Quadrupole time of flight (Q-TOF) and Fourier transform ion cyclotron resonance (FT-ICR) MS techniques are applied to metabolite identification due to their high resolution of pseudomolecular ions. Fragment ions are obtained step-by-step through ion trap (IT) MS, which is helpful for deducing the chemical structures. The acyl groups from fatty acids are confirmed by GC-MS, and neutral fatty acid losses are observed in LC-MS [24].

The fragmentation pathways of different types of* Aconitum* alkaloids include diagnostic ions. For the AC-type of alkaloid, the diagnostic ions are [M+H-18 (water)]^+^, [M+H-60 (acetate from C-8 and C-15)]^+^, [M+H-60-32 (methanol)-28 (carbonyl group)]^+^, and [M+H-60-32-28-122 (benzoic acid at C-14)]^+^[14, 22]. For the BAC-type, the diagnostic ions are [M+H-50 (methanol and water)]^+^, [M+H-50-32]^+^, and [M+H-50-32-18]^+^ [60]. For lipoaconitine, the diagnostic ions are 586 ([Mass of AC+H-60]^+^) with neutral fatty acid losses that correspond to acyl groups at the C-8 position [24].

However, MS^*n*^ analyses only provide a possible fragmentation pattern based on the mass difference between pseudomolecular and fragment ions, and the metabolite confirmations are not necessarily accurate. Considering HA, the demethylation reaction position is ambiguous due to the five methyl groups at the C-1, C-6, C-16, C-18, and nitro positions. Demethylation with dehydrogenation was inferred to occur at the methoxy and hydroxy groups that attach to different skeleton carbons in MA [41] (see Figure 1), while it occurs at the same methoxy group in HA, forming a carbonyl group [43] (see Figure 1). However, detailed structure determination for these two types of metabolites was not provided.

## 7. Conclusions

**Figure 2:**
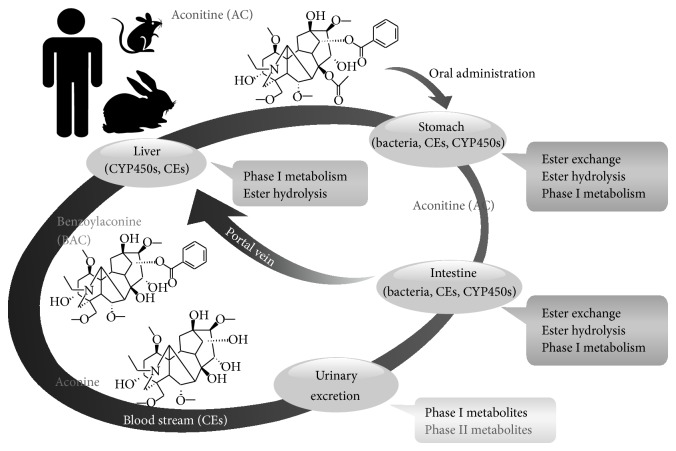
The proposed process of toxicity reduction after oral AC administration in humans and experimental animals. The metabolites from ester exchange are lipo-alkaloids. Ester hydrolysis occurs at the C-8 or/and C-14 position, producing benzoylaconine (BAC) and aconine. Phase I metabolism refers to hydroxylation, deoxylation, dehydrogenation, demethylation, and didemethylation/deethylation. A few phase II metabolites were detected in the urine, including BAC glucuronide and AC sulfate conjugates. Cytochrome P450 enzymes (CYP450s), carboxylesterases (CEs), and enzymes produced by intestinal bacteria are involved in gastrointestinal and hepatic metabolism of aconitine (AC).

In this review, we classify and summarize metabolites of highly toxic DDAs and less toxic MDAs from the gastric and intestinal content, intestinal bacterial juice, hepatic microsomes, blood, and urine from different animal species and humans* in vivo* and* in vitro*. For example, considering AC, which is the most researched toxic DDA, we generalize a process of toxicity reduction in body after oral AC administration for the first time (Figure 2).

The metabolites from ester exchange are lipoalkaloids. Ester hydrolysis occurs at the C-8 or/and C-14 position, producing benzoylaconine (BAC) and aconine. Phase I metabolism refers to hydroxylation, deoxylation, dehydrogenation, demethylation, and didemethylation/deethylation. A few phase II metabolites were detected in the urine, including BAC glucuronide and AC sulfate conjugates. Cytochrome P450 enzymes (CYP450s), carboxylesterases (CEs), and enzymes produced by intestinal bacteria are involved in gastrointestinal and hepatic metabolism of aconitine (AC).

In conclusion, CYP450s, CEs, and enzymes produced by intestinal bacteria are mainly involved in DDA metabolism in both the gastrointestinal tract and liver after oral administration, including hydroxylation, deoxylation, demethylation, dehydrogen, pyrolysis, ester hydrolysis, and ester exchange. Phase II conjugation of DDAs is not the dominant metabolic process and only a few conjugated DDAs are found in the urine. DDA metabolites in the blood are not as various as those in the urine.

Thus far, reports of less toxic MDA metabolism have only been related to hepatic metabolism. Nevertheless, MDAs may share similar metabolic pathways (except ester hydrolysis at the C-8 position) with DDAs in the gastrointestinal tract based on the same DDA and MDA diterpenoid skeletons and similar hepatic metabolism between DDAs and MDAs.

As summarized above, toxic DDAs and MDAs are converted to metabolites that are less toxic or easier to excrete in the gastrointestinal tract and liver after oral administration. However, for drug excretion, few phase II metabolism conjugations are formed, which are the most hydrosoluble metabolites. Further, this detoxification effect is likely restricted due to rapid DDA absorption by the upper gastrointestinal tract.

Although the many available studies on metabolism and toxicity of DDAs and MDAs are helpful, they are insufficient for safe clinical administration of* Aconitum* herbs. Several issues must be further studied and verified. More attention should be paid to metabolism of MDAs because they are not sufficiently safe for clinical use. Due to metabolic interspecific differences, it is more reasonable to apply human recombinant metabolic isozymes or humanized animal models [67] to a human metabolism study. Studies have not confirmed whether the various metabolites detected in the urine are from gastrointestinal and hepatic metabolism via absorption into the blood or from biotransformation in the kidney. Because the metabolites are detected at trace levels, it is difficult to accumulate such metabolites for identification, bioassays, or toxicity studies. However, the changes in bioactivity or toxicity after metabolism are unambiguous.

Based on our conclusions, it is worthwhile to perform an in-depth investigation of the* Aconitum* herbs compatible with other medicines, such as prescription licorice, which is featured in and crucial to clinical application of* Aconitum* herbs in traditional Chinese medicine. To a certain extent, drug-drug interactions are the essence of a drug-drug combination, in which drug metabolism and/or absorption is changed by affecting (inducing or inhibiting) another with respect to metabolic enzymes or/and transporters; thus, drug pharmacological activity or toxicity is consequently affected [12, 13, 67].

## Figures and Tables

**Table 1:** DDA, MDA, and alcohol amine chemical structures.

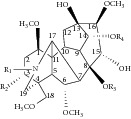						

Compounds	R_1_	R_2_	R_3_	R_4_	Formula	Mass

DDAs						
Aconitine (AC)	Ethyl (Et)	Hydroxy (OH)	Acetyl (Ac)	Benzoyl (Bz)	C_34_H_47_NO_11_	645.3149
Mesaconitine (MA)	Methyl (Me)	OH	Ac	Bz	C_33_H_45_NO_11_	631.2992
Hypaconitine (HA)	Me	Hydrogen (H)	Ac	Bz	C_33_H_45_NO_10_	615.3043
MDAs						
Benzoylaconine (BAC)	Et	OH	H	Bz	C_32_H_45_NO_10_	603.3043
Benzoylmesaconine (BMA)	Me	OH	H	Bz	C_31_H_43_NO_10_	589.2887
Benzoylhypaconine (BHA)	Me	H	H	Bz	C_31_H_43_NO_9_	573.2938
Alcohol amines						
Aconine	Et	OH	H	H	C_25_H_41_NO_9_	499.2781
Mesaconine	Me	OH	H	H	C_24_H_39_NO_9_	485.2625
Hypaconine	Me	H	H	H	C_24_H_39_NO_8_	469.2676

**Table 2:** AC metabolites produced in rabbit stomachs.

DDAs	*m*/*z* (ESI^+^)	Formula	Identification	Neutral loss (Da), identification of fatty acid	Metabolic procedure	MS detection	References
AC	662	C_34_H_47_NO_12_	2′-Hydroxy AC or 3′-AC (M1)^a^	NA^b^	Rabbits and rats; ig,* in vivo*.	IT, FT-ICR	[14]
			3′-Hydroxy AC or 2′-hydroxy AC (M3)^a^				
			4′-Hydroxy AC (M6)^a^				
	632	C_33_H_45_NO_11_	Demethyl AC (M4)	NA			
	630	C_34_H_47_NO_10_	Indaconitine (15-deoxy AC, M5)^c^	NA			
			Deoxyaconitine (3-deoxy AC, M7)				
	618	C_32_H_43_NO_11_	Didemethyl AC or N-deethyl AC (M2)	NA			
	604	C_32_H_45_NO_10_	BAC (hydrolysis product 2)	NA	Rabbits and rats; ig,* in vivo*.	IT, FT-ICR	
	542	C_27_H_43_NO_10_	14-O-Debenzoyl AC (hydrolysis product 1)	NA	Rabbits and rats; ig,* in vivo*.	IT, FT-ICR	
	828	C_47_H_73_NO_11_	8-O-Pentadecanoyl BAC (M10)	242, pentadecanoic acid	Rabbits and rats; ig,* in vivo*.	IT, FT-ICR	
	842	C_48_H_75_NO_11_	8-O-Palmitoyl BAC (M12)	256, palmitic acid			
	864	C_50_H_73_NO_11_	8-O-Linolenoyl BAC (M9)	278, linolenic acid			
	866	C_50_H_75_NO_11_	8-O-Linoleoyl BAC (M11)	280, linoleic acid			
	868	C_50_H_77_NO_11_	8-O-Oleoyl BAC (M13)	282, oleic acid			
	870	C_50_H_79_NO_11_	8-O-Stearoyl BAC (M14)	284, stearic acid			
	978	C_58_H_91_NO_11_	8-O-Hexacosandienoyl BAC (M8)	392, hexacosandienoic acid			

^a^2′, 3′, and 4′, the position in benzoyl group.

^b^Not available.

^c^Deoxy may also be referred to as dehydroxy in the literature.

**Table 3:** Metabolites of AC, MA, and HA converted in intestine.

DDAs	*m*/*z* (ESI^+^)	Formula	Identification	Neutral loss (Da), identification of fatty acid	Metabolic procedure	MS detection	References
AC	662	C_34_H_47_NO_12_	10-Hydroxy AC	NA^a^	Rats; intestinal bacteria; anaerobic incubation at pH 7.0, *in vitro*.	IT	[22] (P4)
	632	C_33_H_45_NO_11_	16-O-Demethyl AC^*^	NA	Rabbits; contents from small intestine and caecum and feces; ig, *in vivo*.	IT	[23] (M3)
					Human; intestinal bacteria; anaerobic incubation, *in vitro*.	IT, FT-ICR	[24] (M1)
	630	C_34_H_47_NO_10_	Indaconitine (15-deoxy AC)^b^		Rabbits; contents from small intestine and caecum and feces; ig, *in vivo*.	IT	[23] (M6)
					Rats; intestinal bacteria; anaerobic incubation at pH 7.0, *in vitro*.	IT	[22] (P5)
			Deoxy AC^*^	NA	Rabbits; contents from small intestine and caecum and feces; ig, *in vivo*.	IT	[23] (M5)
					Human; intestinal bacteria; anaerobic incubation, *in vitro*.	IT, FT-ICR	[24] (M2)
					Rats; intestinal bacteria; anaerobic incubation at pH 7.0, *in vitro*.	IT	[22] (P10)
	616	C_33_H_45_NO_10_	16-O-Demethyl-deoxy AC^*^	NA	Human; intestinal bacteria; anaerobic incubation, *in vitro*.	IT, FT-ICR	[24] (M3)
	604	C_32_H_45_NO_10_	BAC	NA	Rabbits; contents from small intestine and caecum and feces; ig, *in vivo*.	IT	[23] (M2)
					Rats; intestinal bacteria; anaerobic incubation, *in vitro*.^c^	IT	[25]
					Rats; intestinal bacteria; anaerobic incubation, *in vitro*.^d^	IT	[26]
					Rats; intestinal bacteria; anaerobic incubation at pH 7.0, *in vitro*.	IT	[22] (P1)
	590	C_31_H_43_NO_10_	16-O-Demethyl BAC	NA	Rabbits; contents from small intestine and caecum and feces; ig, *in vivo*.	IT	[23] (M1)
	588	C_32_H_45_NO_9_	15-Deoxy BAC	NA	Rats; intestinal bacteria; anaerobic incubation at pH 7.0, *in vitro*.	IT	[22] (P2)
	586	C_32_H_43_NO_9_	Deacetoxy AC	NA	Rats; intestinal bacteria; anaerobic incubation, *in vitro*.^c,d^	IT	[25, 26]
	660	C_35_H_49_NO_11_	8-O-Propionyl BAC	74, propionic acid	Human; intestinal bacteria; anaerobic incubation, *in vitro*.	IT, FT-ICR	[24]
				NA	Rats; intestinal bacteria; anaerobic incubation, *in vitro*.^e^	IT, MALDI source-FT-ICR	[27]
				NA	Rats; intestinal bacteria; anaerobic incubation at pH 7.0, *in vitro*.	IT	[22] (P8)
	674	C_36_H_51_NO_11_	8-O-Butyryl BAC	88, butyric acid	Human; intestinal bacteria; anaerobic incubation, *in vitro*.	IT, FT-ICR	[24]
				NA	Rats; intestinal bacteria; anaerobic incubation, *in vitro*.^e^	IT, MALDI source-FT-ICR	[27]
				NA	Rats; intestinal bacteria; anaerobic incubation at pH 7.0, *in vitro*.	IT	[22] (P9)
	688	C_37_H_53_NO_11_	8-O-Valeryl BAC	102, valeric acid	Human; intestinal bacteria; anaerobic incubation, *in vitro*.	IT, FT-ICR	[24]
				NA	Rats; intestinal bacteria; anaerobic incubation, *in vitro*.^e^	IT, MALDI source-FT-ICR	[27]
	700	C_38_H_53_NO_11_	8-O-Hexenoyl BAC	114, hexenoic acid	Human; intestinal bacteria; anaerobic incubation, *in vitro*.	IT, FT-ICR	[24]
				NA	Rats; intestinal bacteria; anaerobic incubation at pH 7.0, *in vitro*.	IT	[22] (P7)
	690	C_36_H_51_NO_12_	8-O-(3-Hydroxy)-butyryl BAC	NA	Rats; intestinal bacteria; anaerobic incubation at pH 7.0, *in vitro*.	IT	[22] (P11)
	702	C_38_H_55_NO_11_	8-O-Hexanoyl BAC	116, hexanoic acid	Human; intestinal bacteria; anaerobic incubation, *in vitro*.	IT, FT-ICR	[24]
	716	C_39_H_57_NO_11_	8-O-Heptanoyl BAC	130, heptanoic acid	Ibid.	Ibid.	Ibid.
	722	C_40_H_51_NO_11_	8-O-Phenylacetyl BAC	136, phenylacetic acid	Human; intestinal bacteria; anaerobic incubation, *in vitro*.	IT, FT-ICR	[24]
				NA	Rats; intestinal bacteria; anaerobic incubation, *in vitro*.^e^	IT, MALDI source-FT-ICR	[27]
	728	C_40_H_57_NO_11_	8-O-Octenoyl BAC	NA	Rats; intestinal bacteria; anaerobic incubation at pH 7.0, *in vitro*.	IT	[22] (P3)
	736	C_41_H_53_NO_11_	8-O-Phenylpropionyl BAC	150, phenylpropionic acid	Human; intestinal bacteria; anaerobic incubation, *in vitro*.	IT, FT-ICR	[24]
	800	C_45_H_69_NO_11_	8-O-Tridecanoyl BAC	214, tridecanoic acid	Ibid.	Ibid.	Ibid.
	814	C_46_H_71_NO_11_	8-O-Tetradecanoyl BAC	228, tetradecanoic acid	Ibid.	Ibid.	Ibid.
	828	C_47_H_73_NO_11_	8-O-Pentadecanoyl BAC	242, pentadecanoic acid	Ibid.	Ibid.	Ibid.
	842	C_48_H_75_NO_11_	8-O-Palmitoyl BAC	256, palmitic acid	Ibid.	Ibid.	Ibid.
	854	C_49_H_75_NO_11_	8-O-Heptadecenoyl BAC	268, heptadecenoic acid	Ibid.	Ibid.	Ibid.
	856	C_49_H_77_NO_11_	8-O-(Methyl)-palmitoyl BAC	270, methyl palmitic acid	Ibid.	Ibid.	Ibid.
	866	C_50_H_75_NO_11_	8-O-Linoleyl BAC	280, linoleic acid	Human; intestinal bacteria; anaerobic incubation, *in vitro*.	IT, FT-ICR	[24]
				NA	Rats; intestinal bacteria; anaerobic incubation, *in vitro*.^c,d^	IT	[25, 26]
	868	C_50_H_77_NO_11_	8-O-Oleoyl BAC	282, oleic acid	Human; intestinal bacteria; anaerobic incubation, *in vitro*.	IT, FT-ICR	[24]
	870	C_50_H_79_NO_11_	8-O-Stearoyl BAC	284, stearic acid	Ibid.	Ibid.	Ibid.
	882	C_51_H_79_NO_11_	8-O-(9)-Nonadecenoyl BAC	296, nonadecene	Ibid.	Ibid.	Ibid.
	886	C_50_H_79_NO_12_	8-O-(3-Hydroxy)-stearoyl BAC	300, 3-hydroxy stearic acid	Ibid.	Ibid.	Ibid.
	954	C_56_H_91_NO_11_	8-O-Tetracosanoyl BAC	368, tetracosanoic acid	Ibid.	Ibid.	Ibid.
	962	C_57_H_87_NO_11_	8-O-Pentacosatrienoyl BAC	376, pentacosatrienoic acid	Ibid.	Ibid.	Ibid.

MA	590	C_31_H_43_NO_10_	BMA	NA	Rats; intestinal bacteria; anaerobic incubation, *in vitro*.^c,d^	IT	[25, 26]
	572	C_31_H_41_NO_9_	Deacetoxy MA	NA	Ibid.	Ibid.	Ibid.
	660	C_35_H_49_NO_11_	8-O-Butyryl BMA	NA	Rats; intestinal bacteria; anaerobic incubation, *in vitro*.^e^	IT, MALDI source-FT-ICR	[27]
	674	C_36_H_51_NO_11_	8-O-Valeryl BMA	NA	Ibid.	Ibid.	Ibid.
	852	C_49_H_73_NO_11_	8-O-Linoleyl BMA	NA	Rats; intestinal bacteria; anaerobic incubation, *in vitro*.^c,d^	IT	[25, 26]

HA	574	C_31_H_43_NO_9_	BHA	NA	Rats; intestinal bacteria; anaerobic incubation, *in vitro*.^e^	IT, MALDI source-FT-ICR	[27]
					Rats; intestinal bacteria; anaerobic incubation, *in vitro*.^c,d^	IT	[25, 26]
	556	C_31_H_41_NO_8_	Deacetoxy HA	NA	Rats; intestinal bacteria; anaerobic incubation, *in vitro*.^c,d^	IT	[25, 26]
	630	C_34_H_47_NO_10_	8-O-Propionyl BHA	NA	Rats; intestinal bacteria; anaerobic incubation, *in vitro*.^e^	IT, MALDI source-FT-ICR	[27]
	644	C_35_H_49_NO_10_	8-O-Butyryl BHA	NA	Ibid.	Ibid.	Ibid.
	658	C_36_H_51_NO_10_	8-O-Valeryl BHA	NA	Ibid.	Ibid.	Ibid.
	692	C_39_H_49_NO_10_	8-O-Phenylacetyl BHA	NA	Ibid.	Ibid.	Ibid.
	836	C_49_H_73_NO_10_	8-O-Linoleyl BHA	NA	Rats; intestinal bacteria; anaerobic incubation, *in vitro*.^c,d^	IT	[25, 26]

^a^Not available.

^b^Deoxy may also be referred to as dehydroxy in the literature.

^c^DDA was produced through decoction of Aconiti Radix Cocta with Fritillariae Thunbergii Bulbus, Pinelliae Rhizoma Preparatum, and Ampelopsis Radix.

It is not clear whether these compounds were directly metabolized from DDAs or were originally ingested.

^d^DDA was produced through decoction of Aconiti Lateralis Radix Praeparata with Glycyrrhizae Radix and Rhizome as well as with Atractylodis Macrocephalae Rhizoma.

It is not clear whether these compounds were directly metabolized from DDAs or were originally ingested.

^e^In addition to AC and HA monomers, DDAs were also generated from ethyl alcohol extraction ofRadix Aconiti.

It is not clear whether these compounds were directly metabolized from DDAs or were originally ingested.

^*^These metabolites were further biotransformed in the intestine. Metabolites of these intermediate products are listed in Table 4.

**Table 4:** Further biotransformation of intestinal AC metabolites in the intestine.

*m*/*z* (ESI^+^)	Formula	Identification	Neutral loss (Da), identification of fatty acid	Metabolic procedure	MS detection	References
618	C_32_H_43_NO_11_	1,16-Didemethyl AC (M1)	NA^a^	16-O-Demethyl AC (C_33_H_45_NO_11_, 632) from AC; human; intestinal bacteria; anaerobic incubation, *in vitro*.	IT, FT-ICR	[28]
616	C_33_H_45_NO_10_	16-O-Demethyl-3-deoxy AC (M2)^b^	NA			
602	C_32_H_43_NO_10_	1,16-Didemethyl-3-deoxy AC (M3)	NA			
590	C_31_H_43_NO_10_	16-O-Demethyl BAC (M4)	NA			
486	C_24_H_39_NO_9_	16-O-Demethyl aconine (M5)	NA			
646	C_34_H_47_NO_11_	16-O-Demethyl-8-O-propionyl BAC	74, propionic acid			
660	C_35_H_49_NO_11_	16-O-Demethyl-8-O-butyryl BAC	88, butyric acid			
674	C_36_H_51_NO_11_	16-O-Demethyl-8-O-valeryl BAC	102, valeric acid			
		16-O-Demethyl-8-O-(methyl)-butyryl BAC	102, methyl butyric acid			
696	C_38_H_49_NO_11_	16-O-Demethyl-8-O-heptatrienoyl BAC	124, heptatrienoic acid			
698	C_38_H_51_NO_11_	16-O-Demethyl-8-O-heptadienoyl BAC	126, heptadienoic acid			
700	C_38_H_53_NO_11_	16-O-Demethyl-8-O-heptenoyl BAC	128, heptenoic acid			
702	C_38_H_55_NO_11_	16-O-Demethyl-8-O-heptanoyl BAC	130, heptanoic acid			
710	C_39_H_51_NO_11_	16-O-Demethyl-8-O-octatrienoyl BAC	138, octatrienoic acid			
716	C_39_H_57_NO_11_	16-O-Demethyl-8-O-octanoyl BAC	144, octanoic acid			
730	C_40_H_59_NO_11_	16-O-Demethyl-8-O-nonanoyl BAC	158, nonanoic acid			
736	C_41_H_53_NO_11_	16-O-Demethyl-8-O-decatetraenoyl BAC	164, decatetraenoic acid			
762	C_43_H_55_NO_11_	16-O-Demethyl-8-O-dodecapentaenoyl BAC	190, dodecapentaenoic acid			
764	C_43_H_57_NO_11_	16-O-Demethyl-8-O-dodecatetraenoyl BAC	192, dodecatetraenoic acid			
766	C_43_H_59_NO_11_	16-O-Demethyl-8-O-dodecatrienoyl BAC	194, dodecatrienoic acid			
778	C_44_H_59_NO_11_	16-O-Demethyl-8-O-tridecatetraenoyl BAC	206, tridecatetraenoic acid			
786	C_44_H_67_NO_11_	16-O-Demethyl-8-O-(methyl)-dodecanoyl BAC	214, methyl dodecanoic acid			
800	C_45_H_69_NO_11_	16-O-Demethyl-8-O-retradecanoyl BAC	228, tetradecanoic acid			
854	C_49_H_75_NO_11_	16-O-Demethyl-8-O-oleoyl BAC	282, oleic acid			
856	C_49_H_77_NO_11_	16-O-Demethyl-8-O-stearoyl BAC	284, stearic acid			
870	C_50_H_79_NO_11_	16-O-Demethyl-8-O-(methyl)-stearoyl BAC	298, methyl stearic acid			
884	C_51_H_81_NO_11_	16-O-Demethyl-8-O-arachidyl BAC	312, arachidic acid			
898	C_52_H_83_NO_11_	16-O-Demethyl-8-O-heneicosanoyl BAC	326, heneicosanoic acid			
926	C_54_H_87_NO_11_	16-O-Demethyl-8-O-tricosanoyl BAC	354, tricosanoic acid			

616	C_33_H_45_NO_10_	16-O-Demethyl-3-deoxy AC (M1)	NA	3-Deoxy AC (C_34_H_47_NO_10_, 630) from AC; human; intestinal bacteria; anaerobic incubation, *in vitro*.	IT, FT-ICR	[29]
614	C_34_H_47_NO_9_	1,13-Dideoxy AC (M2)	NA			
588	C_32_H_45_NO_9_	3-Deoxy BAC (M3)	NA			
484	C_25_H_41_NO_8_	3-Deoxy aconine (M4)	NA			
644	C_35_H_49_NO_10_	3-Deoxy-8-O-propionyl BAC	74, propionic acid			
658	C_36_H_51_NO_10_	3-Deoxy-8-O-butyryl BAC	88, butyric acid			
700	C_39_H_57_NO_10_	3-Deoxy-8-O-heptanoyl BAC	130, heptanoic acid			
702	C_38_H_55_NO_11_	3-Deoxy-8-O-(2-methyl-3-hydroxy)-valeryl BAC	132, 2-methyl-3-hydroxy valeric acid			
714	C_40_H_59_NO_10_	3-Deoxy-8-O-octanoyl BAC	144, octanoic acid			
730	C_40_H_59_NO_11_	3-Deoxy-8-O-(3-hydroxy)-octanoyl BAC	160, 3-hydroxy octanoic acid			
746	C_43_H_55_NO_10_	3-Deoxy-8-O-undecapentaenoyl BAC	176, undecapentaenoic acid			
762	C_44_H_59_NO_10_	3-Deoxy-8-O-dodecatetraenoyl BAC	192, dodecatetraenoic acid			
786	C_44_H_67_NO_11_	3-Deoxy-8-O-(hydroxy)-dodecanoyl BAC	216, hydroxy dodecanoic acid			
800	C_45_H_69_NO_11_	3-Deoxy-8-O-(hydroxy)-tridecanoyl BAC	230, hydroxy tridecanoic acid			
814	C_46_H_71_NO_11_	3-Deoxy-8-O-(3-hydroxy)-tetradecanoyl BAC	244, hydroxy tetradecanoic acid			
828	C_47_H_73_NO_11_	3-Deoxy-8-O-(hydroxy)-pentadecanoyl BAC	258, hydroxy pentadecanoic acid			
854	C_50_H_79_NO_10_	3-Deoxy-8-O-propionyl BAC	284, stearic acid			

602	C_32_H_43_NO_10_	1,16-O-Didemethyl-3-deoxy AC (M1)	NA	16-O-Demethyl-3-deoxy AC (C_33_H_45_NO_10_, 616) from AC; human; intestinal bacteria; anaerobic incubation, *in vitro*.	IT, FT-ICR	[30]
600	C_33_H_45_NO_9_	16-O-Demethyl-3-deoxy-deoxy AC (M2)	NA			
574	C_31_H_43_NO_9_	16-O-Demethyl-3-deoxy BAC (M3)	NA			
470	C_24_H_39_NO_8_	16-O-Demethyl-3-deoxy aconine (M4)	NA			
630	C_34_H_47_NO_10_	16-O-Demethyl-3-deoxy-8-O-propionyl BAC	74, propionic acid			
644	C_35_H_49_NO_10_	16-O-Demethyl-3-deoxy-8-O-butyryl BAC	88, butyric acid			
696	C_39_H_53_NO_10_	16-O-Demethyl-3-deoxy-8-O-octadienoyl BAC	140, octadienoic acid			
700	C_39_H_57_NO_10_	16-O-Demethyl-3-deoxy-8-O-octanoyl BAC	144, octanoic acid			
702	C_38_H_55_NO_11_	16-O-Demethyl-3-deoxy-8-O-(hydroxy)-heptanoyl BAC	146, hydroxy heptanoic acid			
730	C_40_H_59_NO_11_	16-O-Demethyl-3-deoxy-8-O-(hydroxy)-nonanoyl BAC	174, hydroxy nonanoic acid			
746	C_43_H_55_NO_10_	16-O-Demethyl-3-deoxy-8-O-dodecapentaenoyl BAC	190, dodecapentaenoic acid			
762	C_44_H_59_NO_10_	16-O-Demethyl-3-deoxy-8-O-tridecatetraenoyl BAC	206, tridecatetraenoic acid			
778	C_45_H_63_NO_10_	16-O-Demethyl-3-deoxy-8-O-tetradecatrienoyl BAC	222, tetradecatrienoic acid			

^a^Not available.

^b^Deoxy may also be referred to as dehydroxy in the literature.

**Table 5:** Metabolites of DDAs and MDAs converted in the liver.

Alkaloids	*m*/*z* (ESI^+^)	Formula	Identification	Involved CYP450s	Metabolic procedure	MS detection	References
AC	662	C_34_H_47_NO_12_	Hydroxy AC	CYP3A5, CYP2D6	Human; liver microsomes and recombinant CYP450s; incubation, *in vitro*.	Q-TOF	[35] (M6)
				NA^a^	Rats; liver microsome S_9_ fraction; incubation, *in vitro*.	IT	[36] (M5)
					Guinea pigs and mice; liver microsomes; incubation, *in vitro*.	HRMS, MS^2^	[37] (M6)
	644	C_34_H_45_NO_11_	3-Dehydrogen AC	CYP3A4, CYP3A5	Human; liver microsomes and recombinant CYP450s; incubation, *in vitro*.	Q-TOF	[35] (M5)
				NA	Guinea pigs and mice; liver microsomes; incubation, *in vitro*.	HRMS, MS^2^	[37] (M5)
			Dehydrogen AC	CYP3A, CYP1A1/2	Rats; liver microsomes; incubation, *in vitro*.	IT	[4] (M6)
				NA	Rats; liver microsome S_9_ fraction; incubation, *in vitro*.	IT	[36] (M7)
	632	C_33_H_45_NO_11_	16-O-Demethyl AC	CYP3A, CYP1A1/2	Rats; liver microsomes; incubation, *in vitro*.	IT	[4] (M2)
				CYP3A4, CYP3A5, CYP2D6, CYP2C9	Human; liver microsomes and recombinant CYP450s; incubation, *in vitro*.	Q-TOF	[35] (M2)
				NA	Rats; liver microsome S_9_ fraction; incubation, *in vitro*.	IT	[36] (M6)
				NA	Guinea pigs and mice; liver microsomes; incubation, *in vitro*.	HRMS, MS^2^	[37] (M2)
			O-Demethyl AC	CYP3A, CYP1A1/2	Rats; liver microsomes; incubation, *in vitro*.	IT	[4] (M1)
				CYP3A4, CYP3A5, CYP2C8, CYP2D6	Human; liver microsomes and recombinant CYP450s; incubation, *in vitro*.	Q-TOF	[35] (M1)
				NA	Guinea pigs and mice; liver microsomes; incubation, *in vitro*.	HRMS, MS^2^	[37] (M1)
	630	C_34_H_47_NO_10_	Deoxyaconitine (3-deoxy AC)	NA	Guinea pigs and mice; liver microsomes; incubation, *in vitro*.	HRMS, MS^2^	[37] (M7)
			Deoxy AC	NA	Rats; liver microsome S_9_ fraction; incubation, *in vitro*.	IT	[36] (M8)
	618	C_32_H_43_NO_11_	O-Didemethyl AC	CYP3A, CYP1A1/2	Rats; liver microsomes; incubation, *in vitro*.	IT	[4] (M3)
				CYP2D6, CYP3A5	Human; liver microsomes and recombinant CYP450s; incubation, *in vitro*.	Q-TOF	[35] (M4)
				NA	Rats; liver microsome S_9_ fraction; incubation, *in vitro*.	IT	[36] (M4)
				NA	Guinea pigs and mice; liver microsomes; incubation, *in vitro*.	HRMS, MS^2^	[37] (M3)
			N-Deethyl AC	CYP3A, CYP1A1/2	Rats; liver microsomes; incubation, *in vitro*.	IT	[4] (M4)
				CYP3A4, CYP3A5, CYP2D6, CYP2C9	Human; liver microsomes and recombinant CYP450s; incubation, *in vitro*.	Q-TOF	[35] (M3)
				NA	Rats; liver microsomes; incubation, *in vitro. *	Q-TOF	[38] (M4)
				NA	Rats; liver microsome S_9_ fraction; incubation, *in vitro*.	IT	[36] (M2)
				NA	Guinea pigs and mice; liver microsomes; incubation, *in vitro*.	HRMS, MS^2^	[37] (M4)
	604	C_32_H_45_NO_10_	BAC	CYP3A, CYP1A1/2	Rats; liver microsomes; incubation, *in vitro*.	IT	[4] (M5)
				NA	Rats; liver microsome and S_9_ fraction; incubation, *in vitro*.	Q-Trap	[39]
				NA	Rats; liver microsomes; incubation, *in vitro. *	Q-TOF	[38] (M2)
				NA	Rats; liver microsome S_9_ fraction; incubation, *in vitro*.	IT	[36] (M1)
				NA	Guinea pigs and mice; liver microsomes; incubation, *in vitro*.	HRMS, MS^2^	[37] (M8)
	586	C_32_H_43_NO_9_	Deacetoxy AC^b^	NA	Rats; liver microsome S_9_ fraction; incubation, *in vitro*.	IT	[36] (M3)
	482	C_25_H_39_NO_8_	Dehydrated aconine	NA	Rabbits; liver; ig,* in vivo. *	IT	[40]

MA	648	C_33_H_45_NO_12_	Hydroxy MA	CYP3A4, CYP3A5	Human (male); liver microsomes and recombinant CYP450s; incubation, *in vitro*.	Q-TOF	[41] (M5)
			2-Hydroxy MA	NA	Rats; liver microsomes; incubation, *in vitro. *	Q-TOF, QQQ	[38] (M5)
				CYP3A, CYP2C, CYP2D	Rats; liver microsomes; incubation, *in vitro. *	QQQ; IM	[42] (M5)
	630	C_33_H_43_NO_11_	Dehydrogen MA	CYP3A4, CYP3A5	Human (male); liver microsomes and recombinant CYP450s; incubation, *in vitro*.	Q-TOF	[41] (M4)
				NA	Rats; liver microsomes; incubation, *in vitro. *	Q-TOF, QQQ	[38] (M6)
			3-Dehydrogen MA	CYP3A, CYP2D	Rats; liver microsomes; incubation, *in vitro. *	QQQ; IM	[42] (M2)
	618	C_32_H_43_NO_11_	16-O-Demethyl MA	CYP2C8, CYP3A4, CYP3A5	Human (male); liver microsomes and recombinant CYP450s; incubation, *in vitro*.	Q-TOF	[41] (M2)
				CYP3A	Rats; liver microsomes; incubation, *in vitro. *	QQQ; IM	[42] (M4)
			1-O-Demethyl MA	CYP3A, CYP2C	Rats; liver microsomes; incubation, *in vitro. *	QQQ; IM	[42] (M3)
			18-O-Demethyl MA	CYP3A, CYP2C	Rats; liver microsomes; incubation, *in vitro. *	QQQ; IM	[42] (M6)
			Demethyl MA	CYP2C8, CYP2D6, CYP3A5	Human (male); liver microsomes and recombinant CYP450s; incubation, *in vitro*.	Q-TOF	[41] (M1)
			Demethyl MA	CYP3A4, CYP3A5	Human (male); liver microsomes and recombinant CYP450s; incubation, *in vitro*.	Q-TOF	[41] (M3)
	616	C_32_H_41_NO_11_	Demethyl-dehydrogen MA	CYP3A4, CYP3A5	Human (male); liver microsomes and recombinant CYP450s; incubation, *in vitro*.	Q-TOF	[41] (M6)
			Demethyl-dehydrogen MA	CYP2C8, CYP3A4, CYP3A5	Human (male); liver microsomes and recombinant CYP450s; incubation, *in vitro*.	Q-TOF	[41] (M7, M8)
			Demethyl-dehydrogen MA	CYP2C8, CYP2C9, CYP2D6, CYP3A4, CYP3A5	Human (male); liver microsomes and recombinant CYP450s; incubation, *in vitro*.	Q-TOF	[41] (M9)
	590	C_31_H_44_NO_10_	BMA	NA	Rats; liver microsome and S_9_ fraction; incubation, *in vitro*.	Q-Trap	[39]
				NA	Rats; liver microsomes; incubation, *in vitro. *	Q-TOF, QQQ	[38] (M1)

HA	632	C_33_H_45_NO_11_	MA	CYP3A4, CYP3A5, CYP2C19, CYP2D6, CYP2E1	Human (male); liver microsomes and recombinant CYP450s; incubation, *in vitro*.	Q-TOF	[43] (M8)
				CYP3A, CYP2D, CYP2C, CYP2E1	Rats; liver microsomes; incubation, *in vitro. *	QQQ	[44] (M6)
			2-Hydroxy HA	CYP3A, CYP2C, CYP2D, CYP1A2	Rats; liver microsomes; incubation, *in vitro. *	QQQ	[44] (M4)
			Hydroxy HA	CYP3A4, CYP3A5, CYP2C19, CYP2D6, CYP2E1	Human (male); liver microsomes and recombinant CYP450s; incubation, *in vitro*.	Q-TOF	[43] (M7)
	614	C_33_H_43_NO_10_	15-Dehydrogen HA	CYP3A, CYP2D, CYP2E1	Rats; liver microsomes; incubation, *in vitro. *	QQQ	[44] (M2)
	602	C_32_H_43_NO_10_	16-O-Demethyl HA	CYP3A4, CYP3A5, CYP2C19, CYP2D6, CYP2E1	Human (male); liver microsomes and recombinant CYP450s; incubation, *in vitro*.	Q-TOF	[43] (M2)
			1-O-Demethyl HA	CYP3A, CYP2D, CYP2C	Rats; liver microsomes; incubation, *in vitro. *	QQQ	[44] (M5)
			18-O-Demethyl HA	CYP3A, CYP2C	Rats; liver microsomes; incubation, *in vitro. *	QQQ	[44] (M7)
			Demethyl HA	CYP3A4, CYP3A5, CYP2C8, CYP2C19, CYP2D6, CYP2E1	Human (male); liver microsomes and recombinant CYP450s; incubation, *in vitro*.	Q-TOF	[43] (M1)
			Demethyl HA	CYP3A4, CYP3A5, CYP1A2, CYP2C8, CYP2C19, CYP2D6, CYP2E1	Human (male); liver microsomes and recombinant CYP450s; incubation, *in vitro*.	Q-TOF	[43] (M3)
	600	C_32_H_41_NO_10_	Demethyl-dehydrogen HA	CYP3A4, CYP3A5, CYP2C19, CYP2D6, CYP2E1	Human (male); liver microsomes and recombinant CYP450s; incubation, *in vitro*.	Q-TOF	[43] (M4–M6)
	590	C_31_H_43_NO_10_	2-Hydroxy BHA	CYP3A, CYP2C	Rats; liver microsomes; incubation, *in vitro. *	QQQ	[44] (M1)
	588	C_31_H_41_NO_10_	Didemethyl HA	CYP3A4, CYP3A5, CYP2C19, CYP2D6, CYP2E1	Human (male); liver microsomes and recombinant CYP450s; incubation, *in vitro*.	Q-TOF	[43] (M9, M10)
			Didemethyl HA	CYP3A4, CYP3A5, CYP2C19	Human (male); liver microsomes and recombinant CYP450s; incubation, *in vitro*.	Q-TOF	[43] (M11)
	574	C_31_H_43_NO_9_	BHA	CYP3A, CYP2D	Rats; liver microsomes; incubation, *in vitro. *	QQQ	[44] (M3)
				NA	Rats; liver microsomes; incubation, *in vitro. *	Q-TOF, QQQ	[38] (M3)
				NA	Rats; liver microsome and S_9_ fraction; incubation, *in vitro*.	Q-Trap	[39]

BAC	602	C_32_H_43_NO_10_	Dehydrogen BAC (M1, M2)	CYP3A4, CYP3A5	Human; liver microsomes; incubation, *in vitro*.	Q-TOF	[45]
	590	C_31_H_43_NO_10_	Demethyl BAC (M5)	CYP3A4, CYP3A5, CYP2D6			
			Demethyl BAC (M6)	CYP3A4, CYP3A5			
	588	C_31_H_41_NO_10_	Demethyl-dehydrogen BAC (M3)	CYP3A4, CYP3A5			
	576	C_30_H_41_NO_10_	Deethyl BAC or didemethyl BAC (M7)	CYP3A4, CYP3A5			
	574	C_30_H_39_NO_10_	Didemethyl-dehydrogen BAC or deethyl-dehydrogen BAC (M4)	CYP3A4, CYP3A5			

BMA	606	C_31_H_43_NO_11_	Hydroxy BMA (M8)	CYP3A4, CYP3A5	Human; liver microsomes; incubation, *in vitro*.	Q-TOF	[45]
	588	C_31_H_41_NO_10_	Dehydrogen BMA (M1, M2)	CYP3A4, CYP3A5			
	576	C_30_H_41_NO_10_	Demethyl BMA (M5)	CYP3A4, CYP3A5, CYP2D6, CYP2C8			
			Demethyl BMA (M6, M7)	CYP3A4, CYP3A5			
	574	C_30_H_39_NO_10_	Demethyl-dehydrogen BMA (M3, M4)	CYP3A4, CYP3A5			

BHA	590	C_31_H_43_NO_10_	Hydroxy BHA (M7)	CYP3A4, CYP3A5	Human; liver microsomes; incubation, *in vitro*.	Q-TOF	[45]
			BMA (M8)	CYP3A4, CYP3A5			
	572	C_31_H_41_NO_9_	Dehydrogen BHA (M1, M2)	CYP3A4, CYP3A5			
	560	C_30_H_41_NO_9_	Demethyl BHA (M5)	CYP3A4			
			Demethyl BHA (M4, M6)	CYP3A4, CYP3A5			
	558	C_30_H_39_NO_9_	Demethyl-dehydrogen BHA (M3)	CYP3A4, CYP3A5			
	556	C_30_H_37_NO_9_	Demethyl-didehydrogen BHA (M9)	CYP3A4, CYP3A5			

^a^Not available.

^b^Deacetoxy aconitine may also be referred to as pyroaconitine in the literature.

**Table 6:** A comparison of DDA and MDA metabolites in different metabolic procedures.

Alkaloids	Stomach	Intestine	Liver (CYP450s, phase I metabolism)
DDAs	Ester hydrolysis	Ester hydrolysis commonly occurs at C-8	Ester hydrolysis commonly occurs at C-8
	Hydroxylation at 2′/3′/4′ of the benzoyl group	Hydroxylation at C-10	Hydroxylation at C-2
	Deoxylation at C-3/15	Deoxylation at C-3/15	Deoxylation at C-3/15
	Demethylation at the methoxy group	Demethylation at the methoxy group, often at C-1/6/16 or the N-methyl group	Demethylation at the methoxy group, often at C-1/6/16 or the N-methyl group
	Didemethylation at the methoxy group or deethylation at the N-ethyl group	NA^a^	Didemethylation at the methoxy group or deethylation at the N-ethyl group
	NA	Deacetoxylation (pyrolysis)	Deacetoxylation (pyrolysis)
	NA	NA	Dehydrogenation at C-3/15
	NA	NA	Demethylation at C-1/6/16 or the N-methyl group with dehydrogenation at C-3/15; demethylation with dehydrogenation at the same methoxyl group, O remained as a carbonyl group.
	NA	Demethylation and deoxylation	NA
	Lipoalkaloids via ester exchange at C-8 with long chain fatty acids.	Lipoalkaloids via ester exchange at C-8 with short/long chain fatty acids.	NA

MDAs	NA	NA	Hydroxylation
			Demethylation
			Didemethylation or deethylation
			Dehydrogenation
			Demethylation and (di)dehydrogenation

^a^Not available.

**Table 7:** DDA metabolites detected in the plasma.

DDAs	*m*/*z* (ESI^+^)	Formula	Identification	Metabolic procedure	MS detection	References
AC	604	C_32_H_45_NO_10_	BAC	Mouse; plasma; ig, *in vivo*.	GC/MS	[51]
				Rabbit; plasma; ig, *in vivo*.	IT	[52] (M2)
	590	C_31_H_43_NO_10_	16-O-Demethyl BAC	Rabbit; plasma; ig, *in vivo*.	IT	[52] (M3)
	500	C_25_H_41_NO_9_	Aconine	Rats; plasma; iv,* in vivo*.^a^	Q-Trap	[39]
				Mouse; plasma; ig, *in vivo*.	GC/MS	[51]
				Rabbit; plasma; ig, *in vivo*.	IT	[52] (M4)

MA	590	C_31_H_43_NO_10_	BMA	Rats; plasma; iv,* in vivo*.^a^	Q-Trap	[39]
	486	C_24_H_40_NO_9_	Mesaconine			

HA	574	C_31_H_44_NO_9_	BHA	Rats; plasma; iv,* in vivo*.^a^	Q-Trap	[39]

^a^A mixture of AC, MA, and HA was administered via the tail vein.

**Table 8:** Metabolites of AC, MA, and HA (DDAs) detected in the urine.


DDAs	*m*/*z* (ESI^+^)	Formula	Identification	Metabolic procedure	MS detection	References

AC	780	C_38_H_53_NO_16_	BAC glucuronide conjugate	Rats; ig, *in vivo*.	IT	[54]
	726	C_34_H_47_NO_14_S	AC sulfate conjugate			
	662	C_34_H_47_NO_12_	10-Hydroxy AC	Rats; ig, *in vivo*.	IT	[54]
				Rats; ig, *in vivo*.	IT	[36] (M5)
	644	C_34_H_45_NO_11_	3-Dehydrogen AC	Rats; ig, *in vivo*.	IT	[36] (M7)
	632	C_33_H_45_NO_11_	16-O-Demethyl AC	Rats; ig, *in vivo*.	IT	[54]
				Rats; ig, *in vivo*.	IT	[55] (M2)
				Rabbits; ig, *in vivo*.	IT	[56] (M1)
				Rabbits; iv and ig, *in vivo*.	IT	[48] (M1, found in both iv and ig)
				Rabbits (male and female); ig, *in vivo*.	IT	[57] (M5)
				Human (female); po,* in vivo*.^a^	IT	[58] (M4)
				Rats; ig, *in vivo*.	IT	[36] (M6)
				Rabbits; ig, *in vivo*.	IT	[59] (M1)
				Human (female); po,* in vivo*.^b^	IT	[60] (M7)
			1-O-Demethyl AC	Rats; ig, *in vivo*.	IT	[54]
			6-O-Demethyl AC			
			MA	Rats; ig, *in vivo*.	IT	[55] (M1)
	630	C_34_H_47_NO_10_	Deoxy AC	Rats; ig, *in vivo*.	IT	[54]
				Rats; ig, *in vivo*.	IT	[36] (M8)
	618	C_32_H_43_NO_11_	16-O-Demethyl MA	Rats; ig, *in vivo*.	IT	[55] (M3)
			8-Methoxy BAC	Rats; ig, *in vivo*.	IT	[54]
			1-O-Demethyl MA	Rats; ig, *in vivo*.	IT	[54]
			N-Deethyl AC (M2)	Rats; ig, *in vivo*.	IT	[36]
			O-Didemethyl AC (M4)			
	616	C_33_H_45_NO_10_	1-O-Demethyl-13-deoxy AC	Rats; ig, *in vivo*.	IT	[54]
			Demethyl-deoxy AC	Rabbits; iv and ig, *in vivo*.	IT	[48] (M2, found in ig only)
	606	C_31_H_43_NO_11_	10-Hydroxy BMA	Rats; ig, *in vivo*.	IT	[54]
	604	C_32_H_45_NO_10_	BAC	Rabbits; ig, *in vivo*.	IT	[56] (M2)
				Rats; ig, *in vivo*.	IT	[55] (M4)
				Rabbits (male and female); ig, *in vivo*.	IT	[57] (M2)
				Rabbits; ig, *in vivo*.	IT	[59] (M2)
				Rats; ig, *in vivo*.	IT	[54]
				Human (female); po,* in vivo*.^a^	IT	[58] (M1)
				Human (female); po,* in vivo*.^b^	IT	[60] (M4)
				Rats; ig, *in vivo*.	IT	[36] (M1)
	590	C_31_H_43_NO_10_	16-O-Demethyl BAC	Rabbits; ig, *in vivo*.	IT	[56] (M3)
				Rabbits (male and female); ig, *in vivo*.	IT	[57] (M3)
				Rabbits; ig, *in vivo*.	IT	[59] (M3)
	588	C_32_H_45_NO_9_	3-Deoxy BAC	Rats; ig, *in vivo*.	IT	[54]
	586	C_32_H_43_NO_9_	Pyroaconitine (deacetoxy AC)	Rabbits (male and female); ig, *in vivo*.	IT	[57] (M6, found in male only)
				Rats; ig, *in vivo*.	IT	[54]
				Rats; ig, *in vivo*.	IT	[36] (M3)
	500	C_25_H_41_NO_9_	Aconine	Rabbits; ig, *in vivo*.	IT	[56] (M4)
				Rabbits (male and female); ig, *in vivo*.	IT	[57] (M4)
				Rabbits; ig, *in vivo*.	IT	[59] (M4)
				Rats; ig, *in vivo*.	IT	[54]
	482	C_25_H_39_NO_8_	Dehydrated aconine	Human; po, *in vivo*.^c^	IT	[40]

Alkaloids	*m*/*z* (ESI^+^)	Formula	Identification	Metabolic procedure	MS detection	References

MA	766	C_37_H_51_NO_16_	BMA glucuronide conjugate	Rats; ig, *in vivo*.	IT	[61] (M1)
	648	C_33_H_45_NO_12_	10-Hydroxy MA	Rats; ig, *in vivo*.	IT	[61] (M2)
	618	C_32_H_43_NO_11_	1-O-Demethyl MA	Rats; ig, *in vivo*.	IT	[61] (M3)
			Demethyl MA	Rats; ig, *in vivo*.^d^	TOF	[62] (M10)
	616	C_33_H_45_NO_10_	Deoxy MA	Rats; ig, *in vivo*.	IT	[61] (M4)
	590	C_31_H_43_NO_10_	BMA	Rats; ig, *in vivo*.	IT	[61] (M5)
				Human (female); po,* in vivo*.^a^	IT	[58] (M2)
				Human (female); po,* in vivo*.^b^	IT	[60] (M5)
	468	C_24_H_37_NO_8_	Dehydrated mesaconine	Human; po, *in vivo*.^c^	IT	[40]

HA	602	C_32_H_43_NO_10_	16-O-Demethyl HA	Human (female); po,* in vivo*.^a^	IT	[58] (M5)
				Human (female); po,* in vivo*.^b^	IT	[60] (M8)
	574	C_31_H_43_NO_9_	BHA	Human (female); po,* in vivo*.^a^	IT	[58] (M3)
				Human (female); po,* in vivo*.^b^	IT	[60] (M6)

^a,b^DDA was produced through decoction containing Aconiti and Aconiti Kusnezoffii Radix.

It is not clear whether these compounds were directly metabolized from DDAs or originally ingested.

^c^DDA was produced from a medical liquor containing Aconiti Kusnezoffii Radix.

It is not clear whether these compounds were directly metabolized from DDAs or originally ingested.

^d^DDA was produced from a liquid of crude aconite root decoction via ethanol precipitation.

It is not clear whether these compounds were directly metabolized from DDAs or originally ingested.

## Abbreviations

AC: Aconitine
BAC: 14-Benzoylaconine or 8-O-deacetyl aconitine
BHA: 14-Benzoylhypaconine or 8-O-deacetyl hypaconitine
BMA: 14-Benzoylmesaconine or 8-O-deacetyl mesaconitine
CEs: Carboxylesterases
CYP450s: Cytochrome P450 enzymes
DDAs: Diester diterpenoid alkaloids
FT-ICR: Fourier transform ion cyclotron resonance
HA: Hypaconitine
HLM: Human liver microsomes
IM: Ion mobility
IT: Ion trap
LD_50_: Half-maximally lethal dose
MA: Mesaconitine
MDAs: Monoester diterpenoid alkaloids
MRP: Multidrug resistance-associated protein
MS: Mass spectrometry
NA: Not available
P-gp: P-glycoprotein
QQQ: Triple quadrupole
Q-trap: Quadrupole trap
Q-TOF: Quadrupole time of flight
UGTs: Uridine 5-diphosphate- (UDP-) glucuronosyltransferases.
